# Spinosad: a biorational mosquito larvicide for use in car tires in southern Mexico

**DOI:** 10.1186/1756-3305-5-95

**Published:** 2012-05-19

**Authors:** Carlos F Marina, J Guillermo Bond, José Muñoz, Javier Valle, Nelva Chirino, Trevor Williams

**Affiliations:** 1 Centro Regional de Investigación en Salud Pública - INSP, Tapachula, Chiapas, Mexico; 2 ECOSUR, Tapachula, Chiapas, Mexico; 3 Instituto de Ecología AC, Xalapa, Veracruz, Mexico

## Abstract

**Background:**

Car tires are important habitats for mosquito development because of the high density populations they can harbor and their presence in urban settings. Water in experimental tires was treated with one of three insecticides or an untreated control. Aquatic invertebrates were sampled at weekly intervals. Eggs, larval and pupal samples were laboratory-reared to estimate seasonal fluctuations in *Aedes aegypti* and *Ae. albopictus* abundance.

**Results:**

Spinosad treatments at 1 or 5 ppm (mg a.i./liter) provided 6–8 weeks of effective control of *Ae. aegypti*, *Ae. albopictus*, *Culex quinquefasiatus* and *Cx. coronator* larvae, both in the dry season and the rainy season when mosquito populations increased markedly in southern Mexico. Spinosad continued to provide partial control of larvae for several weeks after initial recolonization of treated tires. The larvicidal performance of VectoBac 12AS (*Bacillus thuringiensis* var. *israelensis*) was relatively poor with one week of complete control of *Aedes* spp. larvae and no discernible control of *Culex* spp., whereas the duration of larvicidal activity of 1% temephos mineral-based granules was intermediate between those of VectoBac and spinosad treatments. Populations of chironomids, ostracods and *Toxorhynchites theobaldi* were generally reduced in spinosad and temephos treatments, but were similar in control and VectoBac treatments.

**Conclusion:**

The present study is the first to report spinosad as an effective larvicide against *Cx. coronator*, which is currently invading the southern United States. These results substantiate the use of spinosad as a highly effective mosquito larvicide, even in habitats such as unused car tires that can represent prolific sources of adult mosquitoes.

## Background

Used car tires are an important habitat for the development of container-dwelling mosquitoes many of which are important vectors of human and wildlife arboviruses. International trade in tires that may contain mosquito immature stages has become an important mechanism for the human-assisted dispersal of some exotic species [1], most notably *Ae. albopictus* that has reached the Americas, Africa and Europe via contaminated car tires [2,3]. Tire storage facilities, recycling plants and discarded tires are therefore likely to represent localized sources of medically-important mosquito species in rural and urban settings. The public health importance of mosquito development in tires is clearly recognized [4]. Tires provide four characteristics that favor the development of container-dwelling mosquitoes: (i) they provide shaded conditions in their interior that is favored for oviposition by many species, (ii) their dark color promotes rapid warming in sunlight that speeds larval development. This can advance the seasonal occurrence of vector species in some regions [5], (iii) their shape tends to collect rainfall, seeds and leaf detritus efficiently providing food resources for developing larvae [6,7], and (iv) tires can remain undisturbed for long periods allowing sequential generations to reach extremely high population densities at some sites [8]. Moreover, the vector potential of certain species depends on the conditions they experience during development. In this respect, mosquitoes that develop in tires may be smaller and more susceptible to infection by viruses from vertebrate hosts than conspecifics that develop in natural habitats [9].

The prevalence of vector borne viruses in the human population is a major public health issue in Mexico and many other parts of Latin America and elsewhere [10-12]. The economic impact of dengue virus alone has been conservatively estimated at US$2.1 billion per year in the Americas, mainly in lost productivity and direct medical attention [13].

In Mexico, the principal vector, *Aedes aegypti*, is sympatric over much of its range with the invasive Asian tiger mosquito, *Ae. albopictus* that is currently invading the country from both northern and southern borders [14,15]. *Ae. albopictus* has been firmly implicated as a vector of dengue and several other mortal arboviruses [16]. As such, the latter species represents a major emerging public health threat in the Americas [17,18]. Other mosquito species of medical importance in this region include *Culex* species, particularly *Cx. quinquefasiatus* and *Cx. coronator.* Both these species have the capacity to disseminate various arboviruses including West Nile virus, an emerging pathogen in the Americas [19].

Vector control programs currently focus on the elimination of larval development habitats, often in combination with the treatment of water sources with larvicides, the success of which requires considerable community participation [20]. These measures are accompanied by intra-domiciliary residual spraying, street-level fogging or aerial application of insecticides during outbreaks of vector borne disease [21].

The principal larvicide used for control of *Ae. aegypti* populations in developing countries is the organophosphate temephos (Abate), that is often applied to potential larval habitats as a mineral granule formulation. The widespread use of this compound has led to the development of resistance in some regions [22,23]. In countries with greater resources available for public health programs, temephos has now been replaced with biological insecticides based on *Bacillus thuringiensis* var. *israelensis* or other biorational agents that are derived from, or mimic, natural substances.

Spinosad is a biorational insecticide produced during the fermentation of an actinomycete. Spinosad is mixture of two spinosyn neurotoxins that are highly toxic to certain orders of insects, including Diptera. However, spinosad has very little toxicity to vertebrates and has recently been approved for use as a mosquito larvicide in human drinking water [24].

Spinosad has been shown to be effective in preventing or reducing the development of immature aquatic stages of important vector species, particularly *Ae. aegypti* and *Ae. albopictusAnopheles gambiaeAn. pseudopunctipennisAn. albimanusCx. pipiens* and *Cx. quinquefasicatus*, among others [25]. Most of these studies have been performed under laboratory conditions; studies on the control of these species in natural habitats are limited in number.

The aim of the present study was to evaluate the efficacy of spinosad as a larvicide in car tire habitats. For this, the performance of spinosad as a larvicide was compared with a Bti-based product, VectoBac, and temephos granules in experimental car tires in an urban environment in southern Mexico.

## Methods

### Insecticides

Spinosad was obtained as a liquid suspension concentrate formulation (Tracer 480SC, Dow Agrosciences LLC, Indianapolis, IN) containing 480 g active ingredient (a.i.)/l. Bti was obtained as a suspension concentrate (VectoBac 12AS, Valent BioSciences Corp., Libertyville, IL) containing 12,000 international toxicity units (ITU)/ml. Temephos was obtained as a generic mineral granular formulation comprising 1% a.i. (wt./wt.) provided by the Secretaria de Salud (Mexican government).

### Field trials in used car tires

These experiments were performed in a tire repair yard (14° 50´ N; 92° 11´ W) surrounded by housing in an urbanized zone in the town of Metapa, Chiapas at an altitude of 100 m above sea level. A total of 75 used car tires were arranged in five rows with 15 tires per row, all in an unshaded location. The distance between tires was 4 m with 7 m distance between adjacent rows, covering a total area of 36 × 60 m. Each tire was perforated to create a 5 cm hole in the wall of the tire. Each tire was tied using a nylon rope that was fixed to a wooden stake 1.2 m in height that ensured that every tire remained upright during the experiment. A 4-liter volume of dechlorinated tap water was poured into each tire at the start of the experiment.

A strip of filter paper (2 cm width × 15 cm length, Whatman No. 2) attached to a wooden spatula was placed resting against the inner side of each tire as an oviposition substrate.

Pre-treatment sampling was performed at weekly intervals. This involved 3 weekly samples taken in the experiment performed during the dry season and 2 weekly samples taken in the experiment performed during the rainy season. In both cases, the sample taken one day prior to the application of experimental treatments was considered as timepoint zero. Prior to each sample, tire water temperature was measured using glass laboratory thermometers (range −30 to 50°C). Ambient air temperature and humidity at the experimental site were measured using a digital thermometer-hygrometer (Sper Scientific, Scottsdale, AZ). All temperature and humidity measurements were performed between 09:00 and 12:00 hrs.

Sampling involved emptying the liquid in each tire through the 5 cm hole in the tire wall. The liquid was poured through a fine nylon mesh net (20 cm diameter and with a pore size of 0.70 × 0.17 mm) into a small bucket. Aquatic insects trapped in the net were immediately placed in a white plastic tray containing water, counted, visually identified to genus, recorded, placed in plastic tubes containing water, labeled and taken to the laboratory in an insulated box. The water from the bucket was examined for the presence of additional arthropods and replaced in the upright tire. Water that had evaporated during the intersample period was replaced with dechlorinated tap water to achieve a total volume of 4 liters. The filter paper oviposition substrate was removed and replaced with a new strip. Filter paper strips with evidence of oviposition were labeled and taken to the laboratory.

One day after the timepoint zero pre-treatment sample had been taken, one of five treatments was applied to 15 tires arranged in a randomized design. The treatments were (i) 1 mg i.a/l spinosad (1 ppm), (ii) 5 mg i.a/l spinosad (5 ppm), (iii) 0.4 g 1% temephos granules, (iv) 50 μl Vectobac AS12; v) untreated water (control). Following the application of each treatment, tires were sampled at weekly intervals for a period of 12 weeks.

The first experiment commenced on 7 March 2007 during the dry season and ended on 13 June 2007 at the beginning of the rainy season. The second experiment started on 25 July and finished on 24 October 2007 that was completely within the period of the rainy season. Appropriate measures were taken to avoid cross-contamination between treatments.

### Laboratory rearing of larvae and eggs collected from tires

Larvae and pupae collected from tire samples, mainly third and fourth instars, were reared in groups (maximum 10 insects/group) in the CRISP (Centro Regional de Investigación en Salud Pública) insectary at 28 ± 2°C in Tapachula, Chiapas, Mexico, and fed *ad libitum* on powdered diet (Laboratory Rodent Diet 5001, PMI Nutrition International, Saint Paul, MN). Adults that developed from these samples were identified to species.

Similarly, paper oviposition strips from tires were individually placed in plastic trays containing dechlorinated tap water in the CRISP insectary. Larvae that emerged from these eggs were reared on powdered diet, allowed to pupate and emerged as adults that were subsequently counted and identified to species. The proportion of hatched eggs was noted for each paper strip.

### Statistical analyses

Numbers of *Aedes* spp. larvae and pupae were summed prior to analysis due to the low numbers of immature insects in some treatments. The same procedure was applied to larvae and pupae of *Culex* spp. and chironomids in separate analyses. Separate mixed models were then fitted using the results from each genus of insects. For this, a compound symmetry covariance structure was specified in SAS (SAS Institute Inc., Cary, NC). To define critical levels of significance Bonferroni correction was applied to all multiple comparison procedures that resulted in α = 0.005. To meet normality assumptions, numbers of eggs oviposited on paper strips required log_e_ (*x* + 0.5) transformation whereas proportions of hatched eggs were arcsine-transformed (arcsin √*p*) prior to analysis of variance with treatment and season (wet *vs*. dry) defined as factors. Numbers of ostracods and *Toxorhynchites theobaldi* immature stages were normalized by log_e_ (y + 0.5) transformation and subjected to multivariate ANOVA (MANOVA). The significance of treatment differences was determined by Tukey test (P < 0.05).

## Results

### Dry season study

The average (±SE) air temperature during the dry season sampling period was 34.9 ± 0.3°C (range 26.8 – 43.7°C) whereas relative humidity averaged 55.3 ± 1.1% (range 35 - 81%). Average tire water temperature was 27.8 ± 0.6°C (range 24–32°C). The average volume of water that evaporated between sampling times was 0.92 ±0.03 L; losses due to evaporation were replaced with dechlorinated water at each sample.

A total of 2,150 *Aedes* spp. larvae + pupae were observed in the three pre-treatment samples compared to 25,417 in the post-treatment samples. In total, 16,548 *Culex* spp. larvae + pupae were observed in pre-treatment sampling compared to 22,284 in post-treatment samples. Very low numbers of *Uranotaenia* spp. (N = 132) and *Limatus* spp. (N = 6) were observed in post-treatment samples but not in pre-treatment samples; these minority species were not considered further. Chironomids were also present in samples: 165 individuals were observed in pre-treatment samples and 11,277 individuals in post-treatment samples. All chironomids appeared to be species of the genus *Chironomus* based on the characteristic red coloration of the larvae.

**Figure 1:**
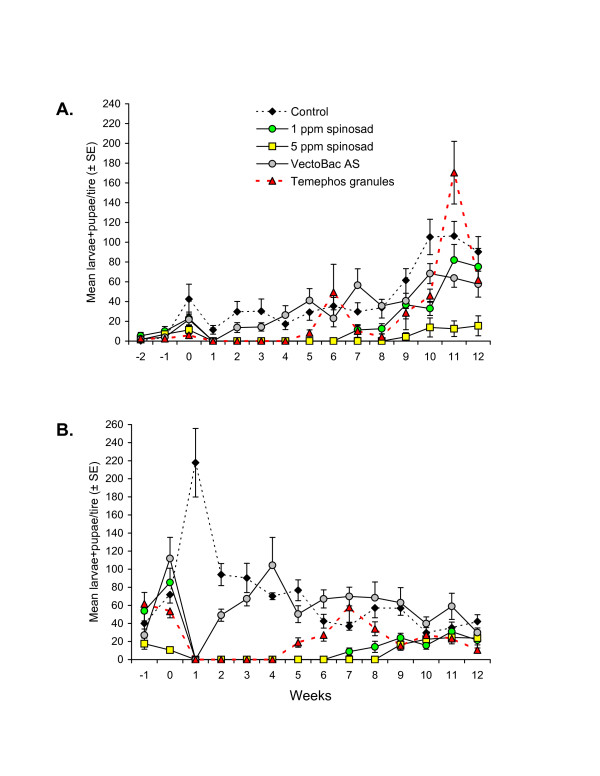
**Mean (±SE) numbers of*Aedes*spp. larvae + pupae observed in car tires sampled at weekly intervals pre- and post-treatment with insecticides in experiments performed in (A) dry season and (B) wet season, in southern Mexico.** For clarity, only half the error bar is shown for some points.

**Figure 2:**
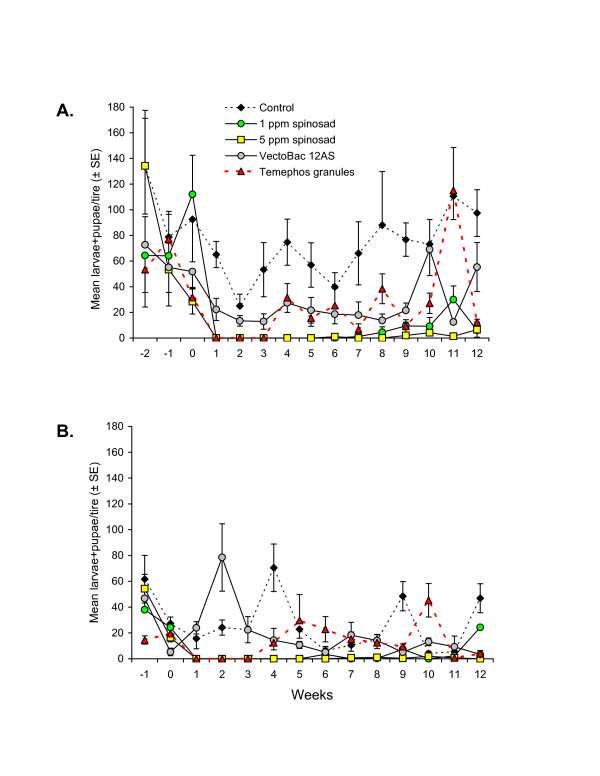
**Mean (±SE) numbers of*Culex*spp. larvae + pupae observed in car tires sampled at weekly intervals pre- and post-treatment with insecticides in experiments performed in (A) dry season and (B) wet season, in southern Mexico.** For clarity, only half the error bar is shown for some points.

**Figure 3:**
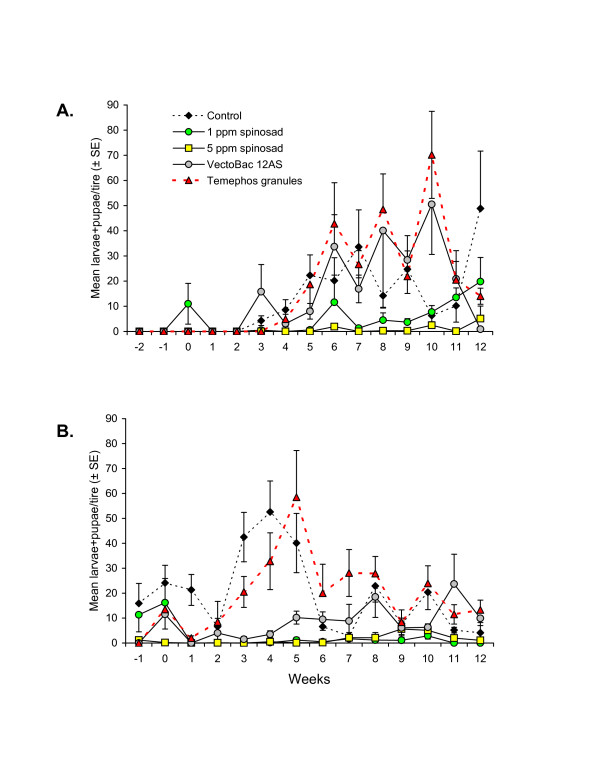
**Mean (±SE) numbers of chiromomid larvae + pupae observed in car tires sampled at weekly intervals pre- and post-treatment with insecticides in experiments performed in (A) dry season and (B) wet season, in southern Mexico.** For clarity, only half the error bar is shown for some points.

Larvae and pupae of *Aedes* spp. were present in all pre-treatment samples and increased in number during the pre-treatment sampling period in all cases (Figure 1A; statistical comparisons between treatments at each sample time shown in Figures 1, 2 and 3 are given in Additional file 1 online: http://www.parasitesandvectors.com). Numbers of *Aedes* spp. in the control treatment fluctuated between 11.4 and 106.3 larvae + pupae/tire in each sample during the 12-week post-treatment period. One week post-treatment the numbers of *Aedes* spp. larvae + pupae was reduced to zero in all treatments except the control (F_4, 70_ = 30.2, *P* < 0.0001).

VectoBac treatment resulted in one week of control, after which numbers of *Aedes* spp. immature stages were similar to that of the control treatment until the end of the trial. The 5 ppm spinosad treatment was significantly more effective at controlling *Aedes* spp. immature stages than any of the other insecticide treatments (F_14, 979_ = 65.2, *P* < 0.0001). Spinosad at concentrations of 1 ppm or 5 ppm provided 6 or 8 weeks of complete control, respectively, after which numbers of *Aedes* spp. were significantly lower than that of the control treatment until 9 or 12 weeks post-treatment, respectively (Figure 1A). Temephos granule treatment resulted in 4 weeks of complete control after which numbers of *Aedes* spp. fluctuated for 5 weeks before consistently reaching numbers similar to that of the control treatment at 10 weeks post-treatment.

Results on the control of *Culex* spp. larvae + pupae during the dry season were similar to those observed for *Aedes* spp. Pre-treatment observations revealed that *Culex* spp. immature stages were present in all treatments and were generally more abundant than *Aedes* spp. immature stages (Figure 2A). Numbers of *Culex* spp. in the control treatment varied between 25 and 110 larvae + pupae per tire during the post-treatment period. VectoBac treatment resulted in a significant but minor reduction in the numbers of *Culex* spp. at 1-week post-treatment, after which numbers remained significantly below control numbers in all samples taken thereafter, except for those samples taken at 2, 8, and 10 weeks post-treatment. The spinosad treatments provided longer lasting control of *Culex* immature stages than VectoBac or temephos treatments. The 1 ppm spinosad treatment provided 6 weeks of absolute control followed by 6 weeks of partial control of *Culex* spp. whereas the 5 ppm spinosad treatment resulted in 8 weeks of complete control followed by 4 weeks of partial control with very low numbers of *Culex* larvae + pupae observed in tires from this treatment. In contrast, the temephos granule treatment resulted in 3 weeks of absolute control after which numbers fluctuated and were often similar to those observed in the control and VectoBac treatments (Figure 2A).

Colonization of tires by chironomids (mostly *Chironomus* spp.) was slow at the start of the dry season experiment. Larvae were only observed in one treatment during the pre-sampling period (Figure 3A). Similarly, chironomids did not colonize the control treatment until 3 weeks post-treatment. VectoBac provided no control of chironomid larvae whereas temephos granules delayed colonization for 1 week compared to the control treatment. In contrast, 1 ppm spinosad treatment resulted in reduced chironomid densities during weeks 4–9 of the dry season experiment compared to control densities. The most effective treatment was 5 ppm spinosad that provided complete protection against chironomid colonization of tires for 5 weeks post-treatment and resulted in very low levels of chironomid larvae + pupae for the remaining 7 weeks of the experiment.

### Rainy season study

The mean air temperature during the rainy season sampling period was 31.2 ± 0.2°C (range 27 – 37°C). Relative humidity varied from 57.7 - 86.8% with a mean value of 68.4 ± 0.8%. The average tire water temperature was 26.2 ± 0.04°C (range 23–32°C). The average volume of water that evaporated from tires between samples was 0.80 ± 0.02 L; evaporational losses were replaced with dechlorinated water at each sample.

A total of 7,999 *Aedes* spp. larvae + pupae were observed during the pre-treatment period compared to 28,064 in the post-treatment samples. In total, 4,620 *Culex* spp. larvae + pupae were observed in pre-treatment sampling compared to 10,579 in post-treatment samples. Very low numbers of *Uranotaenia* spp. (7 specimens in pre-treatment samples and 66 in post-treatment samples) and *Limatus* spp. (79 specimens exclusively in post-treatment samples) were observed and were not considered further. A total of 1,408 chironimids were observed in pre-treatment samples and 9,270 in post-treatment samples. Almost all chironomids were members of the genus *Chironomus*, but were not identified to species.

Immature stages of *Aedes* spp. were registered in all treatments during pre-treatment sampling although significant differences were observed in pre-treatment samples that may be related to variation in the establishment of mosquito populations in the tires (Figure 1B). The numbers of *Aedes* larvae + pupae fluctuated between 29.7 and 217.8 individuals/tire during post-treatment sampling in the control treatment. Application of VectoBac provided 1 week of complete control of *Aedes* spp; samples taken subsequently indicated that this treatment did not provide significant control of *Aedes* immature stages during the period of the trial.

The 5 ppm spinosad treatment was significantly more effective at controlling *Aedes* immature stages than any of the other treatments (F_13, 913_ = 48.1, *P* < 0.0001). The 1 ppm and 5 ppm spinosad treatments resulted in complete control of *Aedes* spp. during 6 and 8 weeks, respectively. This was followed by a period of significantly reduced immature numbers in four of the five subsequent samples in the 1 ppm spinosad treatment and in all four weekly samples taken during the final month of the trial in the 5 ppm spinosad treatment (Figure 1B). Temephos treatment gave complete control of *Aedes* spp. for 4 weeks followed by partial but significant reductions in numbers of immature stages, compared to those registered in the control treatment, in six of the following eight samples.

In the case of *Culex* spp., immature stages were present in all treatments prior to the application of experimental treatments (Figure 2B). Numbers of *Culex* spp. larvae + pupae varied between 4.1 and 70.5 individuals/tire during post-treatment sampling in the control treatment. The VectoBac treatment had no immediate significant effect on numbers of *Culex* spp immature stages, although numbers in 4 out of 12 samples during the post-treatment period were significantly reduced compared to the control treatment.

The 1 ppm spinosad treatment resulted in complete elimination of *Culex* immature stages for 5 weeks post-treatment, after which numbers of larvae + pupae remained very low or were absent until the 12-week sample (Figure 2B). Similarly, the 5 ppm spinosad treatment resulted in a complete absence of *Culex* immature stages for 6 weeks post-treatment followed by very low numbers or absence of members of this genus until the end of the trial. The temephos treatment resulted in 3 weeks of absence of *Culex* spp. in tires. After that time numbers in the temephos treatment were statistically similar or higher than those observed in the control for 5 out of 9 samples, or for 8 out of 9 samples compared with the VectoBac treatment.

Tires were rapidly colonized by chironomids in the wet season experiment; chironomids were observed in all treatments in the zero timepoint sample, immediately prior to the application of each treatment (Figure 3B). Numbers of chironomid larvae + pupae in the control fluctuated between 2.9 and 52.6 during the post-treatment period. VectoBac and temephos granules provided 1 week of control of chironomids and thereafter numbers fluctuated in these treatments but did not differ significantly from those of the control in 6 of the following 11 samples. Both spinosad treatments resulted in significantly reduced chironomid numbers for the 12 week post-treatment period of the experiment, except for one sample in the 5 ppm spinosad treatment taken at 10 weeks post-treatment (Figure 3B).

### Oviposition, egg hatch and laboratory rearing of field collected insects

In the dry season, a total of 7451 eggs of *Aedes* spp. were registered on oviposition traps. Total egg numbers per treatment ranged from 1095 to 1916 (pooled across all samples) but did not differ significantly between treatments (F_4, 70_ = 1.9, *P* = 0.1). Of the eggs reared to adulthood in the laboratory, 53.8% (N = 663) were *Ae. albopictus* and the remainder *Ae. aegypti*.

Significantly higher numbers of eggs were laid in the wet season compared to the dry season (F_1, 144_ = 5.7, *P* = 0.01) with a total of 9581 *Aedes* spp. eggs registered on oviposition traps. Egg numbers on oviposition traps varied from 75.9 ± 10.9 eggs per replicate in the temephos treatment (total 1139) that was significantly reduced compared to the 5 ppm spinosad treatment (96.8 ± 18.0), 1 ppm spinosad (157.3 ± 26.5) VectoBac (182.9 ± 30.6) and control (125.9 ± 21.8) treatments (F_4, 70_ = 3.3, *P* = 0.01).

*Aedes albopictus* was more prevalent in the wet season than in the dry season with 67.9% (N = 1513) of the eggs reared to adulthood in the laboratory, being *Ae. albopictus* and the remainder *Ae. aegypti*. The percentage of egg hatch across all treatments increased significantly from 27.0% (N = 75) in the dry season to 32.6% (N = 75) in the rainy season (F_1,__144_ = 23.4, *P* <0.001). Percentage of egg hatch did not differ significantly between treatments in the dry season (F_4, 70_ = 0.5, *P* = 0.7), whereas in the rainy season egg hatch was significantly higher in the temephos (36.8%), control (33.4%), VectoBac (32.8%) and the 1 ppm spinosad (30.9%) treatments compared to the 5 ppm spinosad treatment (29.2%) (F_4, 70_ = 3.2, *P* = 0.018).

A total of 1681 adult mosquitoes were reared in the insectary from larvae and pupae collected from experimental tires during the dry season, 95.2% of which were identified to species. Of the *Aedes* spp. reared from larvae and pupae collected in the dry season, 52.8% (N = 887) were *Ae. aegytpti* and 37.7% (N = 634) were *Ae. albopictus*. The adult sex ratio was 0.45 male for *Ae. albopictus* and 0.48 male for *Ae. aegypti*. The remaining specimens comprised *Culex coronator* 0.24% (N = 4), *Haemagogus equinus* 4.5% (N = 76) and 80 specimens of *Culex* spp. (4.8%) that were not identified to species. These species were clearly underrepresented in laboratory rearing that was designed specifically to estimate variation in the relative abundance of each of the *Aedes* species present.

Overall, 97.5% of adult mosquitoes reared in the laboratory from samples taken in the wet season were identified to species. Of the *Aedes* spp. reared from field-collected larvae and pupae, 64.3% were *Ae. albopictus* (N = 2533) and the remainder were *Ae. aegypti* (N = 1098), and four specimens of *Ae. podographicus*. The adult sex ratio was 0.49 male in *Ae. albopictus* and 0.51 male in *Ae. aegypti*. Of the four remaining species that were not *Aedes* spp., 1.6% were *Cx. coronator* (N = 65), 0.3% were *Cx. quinquefasciatus (*N = 11*),* 2.5% *Culex* spp. (N = 98), and the remainder 2.6% were *Hg. equinus* (N = 104) or 0.6% *Limatus durhamii* (N = 25), although again, these values are clearly underestimates of the prevalence of these species in tires.

### Effects on non-target organisms

During the dry season experiment a total of 912 non-target organisms were observed during post-treatment sampling. The most abundant predators of mosquito and chironomids were ostracods and the predatory mosquito *Toxorhynchites theobaldi* with 36 and 685 specimens, respectively, registered over the dry season study. Small numbers of coleopterans (N = 10), copepods (N = 6) were also registered, as were other organisms (N = 275), most of which were insects that had likely drowned. Numbers of predatory ostracods and *Tx. theobaldi* differed significantly between treatments (Pillai's F_8, 88_ = 2.501, *P* = 0.01). Predator *Tx. theobaldi* were absent in the 5 ppm spinosad and temephos treatments, whereas ostracod numbers were lowest in the two spinosad treatments during the dry season experiment (Table 1).

**Table 1:** **Mean (±SE) numbers of*Toxorhynchites theobaldi*and ostracods observed in tires following insecticide treatments in the dry and wet season trials**

Treatment	Dry season		Wet season	
	*Tx. theobaldi*	Ostracods	*Tx. theobaldi*	Ostracods
Control	1.3 ± 1.1^a^	23.8 ± 11.9^a^	6.8 ± 2.3^a^	9.7 ± 5.1^a^
VectoBac 12AS	0.4 ± 0.4^ab^	24.5 ± 12.6^a^	2.2 ± 1.3^ab^	43.0 ± 34.5^ab^
1 ppm spinosad	1.3 ± 1.1^a^	2.8 ± 2.3^b^	2.0 ± 1.6^ab^	0.0 ± 0.0^a^
5 ppm spinosad	0.0 ± 0.0^b^	0.0 ± 0.0^b^	1.0 ± 0.4^b^	0.0 ± 0.0^a^
Temephos	0.0 ± 0.0^b^	5.9 ± 2.8^ab^	4.9 ± 1.3^ab^	159.0 ± 67.7^b^

Mean values were pooled across the 12 week period following insecticide treatment.

Values followed by identical letters do not differ significantly for comparisons between treatments within each column (Tukey test, P > 0.05).

Dry season treatment effect, MANOVA, Pillai's trace 0.3705, F_8, 88_ = 2.501, *P* = 0.017.

Wet season treatment effect, MANOVA, Pillai's trace 0.52934, F_8, 88_ = 3.959, *P* < 0.001.

Non-target organisms were markedly more abundant in the wet season compared to the dry season, with a total of 3129 specimens registered in post-treatment sampling in the wet season. The total number of predatory *Tx. theobaldi* was 233 and a total of 2550 ostracods were registered. There were also increased numbers of copeopods (N = 363) compared to the dry season, but few coleopterans (N = 4) and few other insects (N = 9).

Treatment differences in *Tx. theobaldi* and ostracod numbers were highly significant in the wet season (Pillai's F_8, 88_ = 3.959, *P* < 0.0001). Mean numbers of *Tx. theobaldi* were lowest in the 5 ppm spinosad treatment and were similar to the control in the other treatments (Table 1). Unexpectedly, mean numbers of ostracods were highest in the temephos and VectoBac treatments for reasons that are not clear, significantly lower in the control treatment and absent in both the spinosad treatments.

## Discussion

Car tires are important habitats for mosquito development because of the high density populations that they can harbor and their frequent proximity to peridomestic and urban settings. In the present study the predominant mosquito species observed developing in car tires were *Ae. aegyptiAe. albopictusCx. quinquefasiatus*, and *Cx. coronator*. Analysis of tire-inhabiting mosquitoes in the eastern United States noted that studies have reported an average of eight species in each mosquito community, including *Ae. albopictus* among the most commonly reported species in tires in the south-eastern US [4].

The abundance of all the species was seasonally affected; mosquito population densities were approximately twice as high in the wet season compared to the dry season. This difference was particularly marked in *Ae. albopictus* that represented 53.8% (eggs on ovitraps) or 37.7% (laboratory-reared larvae) of the *Aedes* populations in the dry season compared to 67.9% or 64.3% of the *Aedes* populations during the rainy season, based on insects reared from ovitraps or laboratory-rearing of field-collected larvae, respectively. Similar seasonal differences in the dominance of *Ae. albopictus* populations over those of *Ae. aegypti* have been reported from tire habitats in Brazil [26] and cemetery habitats in Mexico [27].

Spinosad was clearly the most efficient larvicide tested with absolute or near absolute control of developing *Aedes* spp. and *Culex* spp. larvae for periods of 6–8 weeks depending on season and concentration. The 5 ppm spinosad treatment provided complete larvicial activity for between one and two weeks longer than the 1 ppm spinosad treatment. The duration of the control period was broadly similar during the wet and dry seasons, although it was not possible to test this formally as experiments were not replicated within seasons. Nonetheless, this observation indicates that the dilution of spinosad by rainfall during the wet season was unlikely to be a significant factor affecting the residual toxicity of this product in experimental tires. Previous studies by us [28] suggest that exposure to sunlight was likely to have been a significant factor affecting the duration of larvicidal activity, given that the half life of spinosad in an aquatic environment has been estimated at <3 days when directly exposed to solar ultraviolet radiation [28]. Despite this, spinosad continued to exhibit partial larvicidal activity for several weeks after larvae were first observed to have recolonized treated tires. It is also important to note that the present study is the first to report effective control of *Cx. coronator* by spinosad in habitats where this species is common but see [27], although the relative abundance of this species could not be estimated from laboratory-reared samples that were designed to estimate the variation in the *Aedes* species present in tires. The neotropical *Cx. coronator* is now attracting attention as it rapidly invades temperate areas of the southern United States [29].

The period of larvicidal activity was similar between *Aedes* and *Culex* mosquitoes, suggesting that both these genera are broadly similar in their susceptibility to this product. Analysis of published studies on laboratory-based concentration-mortality metrics suggested no systematic differences in susceptibility to spinosad according to genus [25]. According to this analysis *Cx. quinquefasciatus* and *Ae. aegypti* were of intermediate susceptibility whereas the susceptibility of *Ae. albopictus* was reported to differ markedly in different studies.

Spinosad has been found to eliminate or dramatically reduce numbers of immature aquatic stages of *Ae. aegypti* and *Ae. albopictus* in cemetery water containers in Mexico [27,28], *Ae. aegypti* in water jars in Thailand [30], *Cx. pipiens* in septic tanks in Turkey [31] or *Cx. pusillusCx. pipiens* and *Aedes caspius* in flooded fields in Egypt [32], *Cx. quinquefasiatus* in field microcosms in California [33], or cesspits, street drains, and disused wells in India [34], *Cx. pipiens pipiensCx. restuans*, and *Ae. japonicus* in catchbasins in Connecticut [35], *Psorophora columbiae* in rice plots in Florida [36], and *An. stephensi* in water tanks in India [37]. Spinosad was also successfully used alone, or in mixtures with an insect growth regulator, for control of insecticide-resistant *Ae. aegypti* populations in Martinique [38,39]. From this, it is clear that relatively few field studies have been performed to date using spinosad, even with mosquito species of major public health performance.

The larvicidal performance of VectoBac was relatively poor with one week of complete control of *Aedes* spp. larvae in each season and no discernible control of *Culex* spp. The low persistence of this Bti-based product, also reported in previous trials, particularly when exposed to direct sunlight [40-42], underscores the need to employ sustained release formulations of Bti to achieve more than fleeting control of mosquito larvae [43].

Temephos was intermediate in the duration of larvicidal performance between Bti and spinosad. Temephos granules provided absolute control of *Aedes* spp. for approximately one week longer than that of *Culex* spp. However, temephos did not provide the long periods of control reported elsewhere [44,45], for reasons that are unclear.

In a recent smaller-scale study [46], the efficacy of a tablet formulation of spinosad (Natular DT, Clarke Mosquito Control Products Inc., Roselle, IL) was tested against that of 1% temephos granules for control of tire-dwelling mosquitoes in northern Mexico on the border with the United States. Spinosad treatment at an estimated rate of 5.25 pm a.i. performed as well as temephos for control of *Aedes* spp. and *Cx. quinquefasiatus* during a 98 day fall and winter period when mosquito populations were very low.

Chironomids were highly sensitive to spinosad although the chironomid fauna of Mexico is very poorly described and only four species have been reported from the state of Chiapas (S. Ibáñez, pers. comm.), arguably the most biodiverse Mexican state. Chironomid densities remained low in spinosad treatments, compared to the control or other treatments, for most of the duration of the study. Laboratory assays have demonstrated spinosad toxicity to a chironomid species of agricultural importance [47], and previous trials reported between 7 and 22 weeks of complete control of chironomids in oviposition traps, depending on treatment concentration and season [48]. It was clear that temephos and VectoBac only briefly affected chironomid populations present in tires. These results suggest that spinosad also merits evaluation for the control of biting midges (Ceratopogonidae) given its high toxicity to members of this family.

Ostracods are common inhabitants of tire habitats [6], and together with *Tx. theobaldi*, were the most abundant non-target fauna in experimental car tires. These taxa were not affected by VectoBac treatment due to the high specificity of the bacterial endotoxins, but were reduced in the presence of spinosad or temephos residues. Whether this was due to the toxicity characteristics of these insecticides or due to the reduced numbers of potential mosquito and chironomid prey items present in spinosad and temephos treatments is unclear and requires laboratory toxicity studies. In contrast, *Toxorhynchites* sp. larvae were observed in the control and spinosad-treated tires on about half of the sample dates although a quantitative analysis was not performed [46]. Temephos treatments have been reported to adversely affect crustacean and benthic macroinvertebrate populations [49-52], reflecting the broad spectrum of insecticidal activity of this compound. In line with its selective ecotoxicological profile, spinosad demonstrated no significant toxicity to a range of aquatic insects in laboratory tests, with the exception of plecopteran species [53]. The susceptibility of daphnids to spinosad varies widely [54], although *Daphnia pulex* was clearly less susceptible to spinosad than to the organophosphate diazinon [55].

## Conclusion

Spinosad treatments provided effective lasting control of *Ae. aegypti*, *Ae. albopictus*, *Cx. quinquefasiatus* and *Cx. coronator* in experimental car tires in an urban setting in southern Mexico, both in the dry season and the rainy season when populations of these mosquitoes increased markedly. In this sense, spinosad outperformed VectoBac and temephos granule treatments that provided brief or intermediate periods of control. Populations of other organisms, notably chironomids, ostracods and the predatory mosquito *Tx. theobaldi* were also reduced in spinosad and temephos treatments. The results of this study contribute to a growing literature indicating that spinosad is a highly effective larvicide against mosquitoes in urban areas, where vector control measures are a key component of public health programs.

## Abbreviations

ppm, Parts per million; a.i., Active ingredient.

## Competing interests

The authors declare that they have no competing interests.

## Authors' contributions

CFM obtained funding via a competitive proposal. JGB, JM, NC, CFM performed field studies. JM, NC performed laboratory rearing. JV performed the statistical analyses. CFM, JGB, JV, TW designed the study, TW, CFM wrote the manuscript. All authors read and approved the final version of the manuscript.

## Author details

^1^Centro Regional de Investigación en Salud Pública - INSP, 19 Pte. esq. 4 Ave. Norte, Tapachula, Chiapas 30700, Mexico; ^2^El Colegio de la Frontera Sur (ECOSUR), Aptdo Postal 36, Tapachula, Chiapas 30700, Mexico, ^3^Instituto de Ecología AC (INECOL), Aptdo Postal 63, Xalapa, Veracruz 91070, Mexico.

## Supplementary Material

Additional file 1 Parasites & Vectors (Supplemental material online). Click here for file

## References

[B1] Nawrocki SJ Craig GB Further extension of the range of the rock pool mosquito, Aedes atropalpus, via tire breeding J Am Mosq Control Assoc 1989 5 110 114 2708986

[B2] Hawley WA Reiter P Copeland RS Pumpuni CB Craig GB Aedes albopictus in North America: probable introduction in used tires from northern Asia Science 1987 236 1114 1116 10.1126/science.3576225 3576225

[B3] Reiter P Sprenger D The used tire trade: a mechanism for the worldwide dispersal of container breeding mosquitoes J Am Mosq Control Assoc 1987 3 494 501 2904963

[B4] Yee DA Tires as habitats for mosquitoes: a review of studies within the eastern United States J Med Entomol 2008 45 581 593 10.1603/0022-2585(2008)45[581:TAHFMA]2.0.CO;2 18714856

[B5] Haramis LD Aedes triseriatus, a comparison of density in tree holes vs. discarded tires Mosq News 1984 44 485 489

[B6] Kling LJ Juliano SA Yee DA Larval mosquito communities in discarded vehicle tires in a forested and unforested site: detritus type, amount and water nutrient differences J Vector Ecol 2007 32 207 217 10.3376/1081-1710(2007)32[207:LMCIDV]2.0.CO;2 18260510 PMC2579933

[B7] Yee DA Kneitel JM Juliano SA Environmental correlates of abundances of mosquito species and stages in discarded vehicle tires J Med Entomol 2010 47 53 62 10.1603/033.047.0107 20180308 PMC3377495

[B8] Pumpuni CB Walker ED Population size and survivorship of adult Aedes triseriatus in a scrap tireyard in northern Indiana J Am Mosq Control Assoc 1989 5 166 172 2746203

[B9] Paulson SL Hawley WA Effect of body size on the vector competence of field and laboratory populations of Aedes triseriatus for La Crosse virus J Am Mosq Control Assoc 1991 7 170 175 1895074

[B10] Hotez PJ Bottazzi ME Franco-Paredes C Ault SK Periago MR The neglected tropical diseases of latin america and the caribbean: a review of disease burden and distribution and a roadmap for control and elimination PLoS Negl Trop Dis 2008 2 e300 10.1371/journal.pntd.0000300 18820747 PMC2553488

[B11] Gubler DJ Atkinson PW The global threat of emergent/re-emergent vector-borne diseases Vector Biology, Ecology and Control 2010 Springer, Netherlands 39 62

[B12] Braks M van der Giessen J Kretzschmar M van Pelt W Scholte EJ Reusken C Zeller H van Bortel W Sprong H Towards an integrated approach in surveillance of vector-borne diseases in Europe Parasit Vectors 2011 4 192 10.1186/1756-3305-4-192 21967706 PMC3199249

[B13] Shepard DS Coudeville L Halasa YA Zambrano B Dayan GH Economic impact of dengue illness in the Americas Am J Trop Med Hyg 2011 84 200 207 10.4269/ajtmh.2011.10-0503 21292885 PMC3029168

[B14] Ibáñez-Bernal S Briseño B Mutebi JP Argot E Rodríguez G Martínez-Campos C Paz R de la Fuente-San Román P Tapia-Conyer R Flisser A First record in America of Aedes albopictus naturally infected with dengue virus during the 1995 outbreak at Reynosa, Mexico Med Vet Entomol 1997 11 305 309 10.1111/j.1365-2915.1997.tb00413.x 9430106

[B15] Casas-Martinez M Torres-Estrada JL First evidence of Aedes albopictus (Skuse) in southern Chiapas in Mexico Emerg Infect Dis 2003 9 606 607 10.3201/eid0905.020678 12737750 PMC2972768

[B16] Paupy C Delatte H Bagny L Corbel V Fontenille D Aedes albopictus, an arbovirus vector: from the darkness to the light Microbes Infect 2009 11 1177 1185 10.1016/j.micinf.2009.05.005 19450706

[B17] Gratz NG Critical review of the vector status of Aedes albopictus Med Vet Entomol 2004 18 215 227 10.1111/j.0269-283X.2004.00513.x 15347388

[B18] Lambrechts L Scott TW Gubler DJ Consequences of the expanding global distribution of Aedes albopictus for dengue virus transmission PLoS Negl Trop Dis 2010 4 e646 10.1371/journal.pntd.0000646 20520794 PMC2876112

[B19] CDC website West Nile virus [http://www.cdc.gov/ncidod/dvbid/westnile/mosquitospecies.htm]

[B20] Heintze C Velasco Garrido M Kroeger A What do community-based dengue control programmes achieve? A systematic review of published evaluations Trans R Soc Trop Med Hyg 2007 101 317 325 10.1016/j.trstmh.2006.08.007 17084427

[B21] WHO Dengue: guidelines for diagnosis, treatment, prevention and control. WHO/HTM/NTD/DEN/2009.1 2009 World Health Organization, Geneva, Switzerland 23762963

[B22] Nauen R Insecticide resistance in disease vectors of public health importance Pest Manag Sci 2007 63 628 633 10.1002/ps.1406 17533649

[B23] Lima EP Paiva MHS de Araújo AP da Silva EVG da Silva UM de Oliveira LN Santana AEG Barbosa CN de Paiva Neto CC Goulart MOF Wilding CS Ayres CFJ de Melo Santos MAV Insecticide resistance in Aedes aegypti populations from Ceará, Brazil Parasit Vectors 2011 4 5 10.1186/1756-3305-4-5 21226942 PMC3035027

[B24] WHO Spinosad DT in drinking-water: use for vector control in drinking-water sources and containers. WHO/HSE/WSH/10.01/12 2010 World Health Organization, Geneva, Switzerland

[B25] Hertlein MB Mavrotas C Jousseaume C Lysandrou M Thompson GD Jany W Richie SA A review of spinosad as a natural mosquito product for larval mosquito control J Amer Mosq Contr Assoc 2010 26 67 87 10.2987/09-5936.1 20402353

[B26] Alves-Honório N Cabello PH Codeço CT Lourenço-de-Oliveira R Preliminary data on the performance of Aedes aegypti and Aedes albopictus immatures developing in water-filled tires in Rio de Janeiro Mem Inst Oswaldo Cruz 2006 101 225 228 10.1590/S0074-02762006000200017 16830718

[B27] Marina CF Bond JG Casas M Muñoz J Orozco A Valle J Williams T Spinosad as an effective larvicide for control of Aedes albopictus and Aedes aggypti, vectors of dengue in southern Mexico Pest Man Sci 2011 67 114 121 10.1002/ps.2043 21162151

[B28] Pérez CM Marina CF Bond JG Rojas JC Valle J Williams T Spinosad, a naturally-derived insecticide, for control of Aedes aegypti: efficacy, persistence and elicited oviposition response J Med Entomol 2007 44 631 638 10.1603/0022-2585(2007)44[631:SANDIF]2.0.CO;2 17695018

[B29] Gray KM Burkett-Cadena ND Eubanks MD Distribution expansion of Culex coronator in Alabama J Am Mosq Control Assoc 2008 24 585 587 10.2987/08-5778.1 19181069

[B30] Thavara U Tawatsin A Asavadachanukorn P Mulla MS Field evaluation in Thailand of spinosad, a larvicide derived from Saccharopolyspora spinosa (Actinomycetales) against Aedes aegypti (L.) larvae SE Asian J Trop Med, Public Health 2009 40 235 242 19323007

[B31] Cetin H Yanikoglu A Cilek JE Evaluation of the naturally-derived insecticide spinosad against Culex pipiens L. (Diptera: Culicidae) larvae in septic tank water in Antalya, Turkey J Vect Ecol 2005 30 151 154 16007970

[B32] Bahgat IM El Kady GA Temerak SA Lysandrou M The natural bio-insecticide spinosad and its toxicity to combat some mosquito species in Ismailia Governorate, Egypt World J Agric Sci 2007 3 396 400

[B33] Jiang Y Mulla MS Laboratory and field evaluation of spinosad, a biorational natural product, against larvae of Culex mosquitoes J Am Mosq Control Assoc 2009 25 456 466 10.2987/Moco-09-5925.1 20099593

[B34] Sadanandane C Boopathi-Doss PS Jambulingam P Zaim M Efficacy of two formulations of the bioinsecticide spinosad against Culex quinquefasciatus in India J Am Mosq Control Assoc 2009 25 66 73 10.2987/08-5807.1 19432070

[B35] Anderson JF Ferrandino FJ Dingman DW Main AJ Andreadis TG Becnel JJ Control of mosquitoes in catch basins in Connecticut with Bacillus thuringiensis israelensis, Bacillus sphaericus, and spinosad J Am Mosq Control Assoc 2011 27 45 55 10.2987/10-6079.1 21476447

[B36] Allen RA Lewis CN Meisch MV Residual efficacy of three spinosad formulations against Psorophora columbiae larvae in small rice plots J Am Mosq Control Assoc 2010 26 116 118 10.2987/09-0014.1 20402361

[B37] Prabhua K Murugana K Nareshkumarb A Badeeswarana S Larvicidal and pupicidal activity of spinosad against the malarial vector Anopheles stephensi Asian Pacif J Trop Med 2011 4 610 613 10.1016/S1995-7645(11)60157-0 21914537

[B38] Darriet F Marcombe S Etienne M Yébakima A Agnew P Yp-Tcha MM Corbel V Field evaluation of pyriproxyfen and spinosad mixture for the control of insecticide resistant Aedes aegypti in Martinique (French West Indies) Parasit Vectors 2010 3 88 10.1186/1756-3305-3-88 20843383 PMC2945330

[B39] Marcombe S Darriet F Agnew P Etienne M Yp-Tcha MM Yébakima A Corbel V Field efficacy of new larvicide products for control of multi-resistant Aedes aegypti populations in Martinique (French West Indies) Am J Trop Med Hyg 2011 84 118 126 10.4269/ajtmh.2011.10-0335 21212213 PMC3005507

[B40] Mulligan FS Schaefer CH Wilder WH Efficacy and persistence of Bacillus sphaericus and B. thuringiensis H. 14 against mosquitoes under laboratory and field conditions J Econ Entomol 1980 73 684 688

[B41] Kramer VL Efficacy and persistence of Bacillus sphaericus, Bacillus thuringiensis var. israelensis, and methoprene against Culiseta incidens (Diptera: Culicidae) in tires J Econ Entomol 1990 83 1280 1285 1976658 10.1093/jee/83.4.1280

[B42] Batra CP Mittal PK Adak T Control of Aedes aegypti breeding in desert coolers and tires by use of Bacillus thuringiensis var. israelensis formulation J Amer Mosq Control Assoc 2000 16 321 323 11198918

[B43] Lacey LA Urbina MJ Heitzman CM Sustained release formulations of Bacillus sphaericus and Bacillus thuringiensis (H-14) for control of container breeding Culex quinquefasciatus Mosq News 1984 44 26 32

[B44] Beehler JW Quick TC DeFoliart GR Residual toxicity of four insecticdes to Aedes triseriatus in scrap tires J Am Mosq Control Assoc 1991 7 121 122 2045803

[B45] Morris CD Dame DA Robinson JW Control of Aedes albopictus in waste tire piles with reduced rates of temephos-treated granules J Am Mosq Control Assoc 1996 12 472 476 8887227

[B46] Garza-Robledo AA Martínez-Perales JF Rodríguez-Castro VA Quiroz-Martínez H Effectiveness of spinosad and temephos for the control of mosquito larvae at a tire dump in Allende, Nuevo Leon, Mexico J Am Mosq Control Assoc 2011 27 404 407 10.2987/11-6133.1 22329273

[B47] Stevens MM Helliwell S Hughes PA Toxicity of Bacillus thuringiensis var. israelensis formulations, spinosad, and selected synthetic insecticides to Chironomus tepperi larvae J Am Mosq Control Assoc 2005 21 446 450 10.2987/8756-971X(2006)21[446:TOBTVI]2.0.CO;2 16506570

[B48] Bond JG Marina CF Williams T The naturally-derived insecticide spinosad is highly toxic to Aedes and Anopheles mosquito larvae Med Vet Entomol 2004 18 50 56 10.1111/j.0269-283X.2004.0480.x 15009445

[B49] Yap HH Lau BL Leong YP Laboratory and field tests of temephos (Abate) on mosquito larvae and non-target organisms in rice fields in Malaysia SE Asian J Trop Med Public Health 1982 13 646 653 6189198

[B50] Fortin C Marie A Leclair R The residual effect of temephos (Abate 4-E) on nontarget communities J Am Mosq Control Assoc 1987 3 282 288 2462615

[B51] Brown MD Watson TM Green S Greenwood JG Purdie D Kay BH Toxicity of insecticides for control of freshwater Culex annulirostris (Diptera: Culicidae) to the nontarget shrimp, Caradina indistincta (Decapoda: Atyidae) J Econ Entomol 2000 93 667 672 10.1603/0022-0493-93.3.667 10902314

[B52] Leveque C Bordeau P, Haines JA, Klein W, Krishna Murti CR The use of insecticides in the onchocerciasis control programme and aquatic monitoring in West Africa Ecotoxicology and Climate 1989 Wiley, Chichester, UK 317 335

[B53] Infante-Rodríguez DA Novelo-Gutiérrez R Mercado G Williams T Spinosad toxicity to Simulium spp. larvae and associated aquatic biota in a coffee-growing region of Veracruz State, Mexico J Med Entomol 2011 48 570 576 10.1603/ME10099 21661318

[B54] Deardorff AD Stark JD Acute toxicity and hazard assessment of spinosad and R-11 to three cladoceran species and Coho salmon Bull Environ Contam Toxicol 2009 82 549 553 10.1007/s00128-009-9643-6 19159051

[B55] Stark JD Vargas RI Demographic changes in Daphnia pulex (Leydig) after exposure to the insecticides spinosad and diazinon Ecotoxicol Environ Safety 2003 56 334 338 10.1016/S0147-6513(02)00074-X 14575672

