# Supplementary material for: Spinosad: a biorational mosquito larvicide for use in car tires in southern Mexico
Source: Parasit Vectors. 2012 May 19;5:95. doi: 10.1186/1756-3305-5-95 (PMC3407511; doi:10.1186/1756-3305-5-95)
Supplement: Additional file 1 — Parasites & Vectors (Supplemental material online). [file 1756-3305-5-95-S1.pdf]

Table 1. Mean (±SE) numbers of *Aedes* spp. larvae + pupae observed in tires at weekly intervals pre- and post-treatment with insecticides in (A) dry and (B) wet season experiments in southern Mexico.

Sample (weeks)	Treatment				
	Control	VectoBac	1 ppm spinosad	5 ppm spinosad	Temephos
(A) Dry season trial					
-2	0.3±0.2[a]	2.3±2.1[a]	5.4±3.4[a]	1.5±1.0[a]	2.7±2.6[a]
-1	3.8±1.8[a]	3.8±1.7[a]	10.0±4.8[a]	6.9±3.3[a]	2.5±1.3[a]
0	42.4±15.2[a]	21.8±5.6[a]	22.9±6.5[a]	11.3±4.3[b]	5.9±2.3[bc]
1	11.4±4.4[a]	0.0±0.0[b]	0.0±0.0[b]	0.0±0.0[b]	0.0±0.0[b]
2	29.7±10.4[a]	13.7±5.0[a]	0.0±0.0[b]	0.0±0.0[b]	0.0±0.0[b]
3	30.2±12.4[a]	14.3±4.2[a]	0.0±0.0[b]	0.0±0.0[b]	0.0±0.0[b]
4	17.3±5.7[a]	26.3±9.5[a]	0.0±0.0[b]	0.0±0.0[b]	0.0±0.0[b]
5	29.1±8.1[a]	41.0±12.0[a]	0.0±0.0[c]	0.0±0.0[c]	8.3±3.0[b]
6	35.5±8.6[a]	23.0±8.6[a]	0.0±0.0[b]	0.0±0.0[b]	49.3±28.4[a]
7	29.7±9.1[a]	56.5±16.7[a]	11.3±4.3[b]	0.0±0.0[c]	10.2±6.4[b]
8	33.8±10.5[a]	35.6±6.6[a]	12.5±5.1[b]	0.0±0.0[c]	4.4±2.7[b]
9	61.5±11.8[a]	40.7±7.7[a]	36.8±25.3[ab]	4.3±4.1[c]	28.5±6.0[b]
10	105.3±17.9[a]	68.3±10.1[a]	33.0±7.1[b]	13.8±9.6[c]	45.9±6.7[ab]
11	106.3±14.8[a]	63.7±9.2[ab]	81.9±15.9[b]	12.5±7.9[c]	170.4±31.7[a]
12	90.3±15.4[a]	57.6±13.1[a]	75.2±18.5[a]	15.5±10.1[b]	61.8±9.0[a]
(B) Wet season trial					
-1	40.1±11.2[a]	27.1±6.7[b]	53.9±11.1[a]	17.6±6.2[b]	61.6±12.7[a]
0	71.8±9.2[a]	111.8±23.5[a]	85.3±15.8[a]	10.6±3.2[c]	53.5±7.3[b]
1	217.8±37.9[a]	0.0±0.0[b]	0.0±0.0[b]	0.0±0.0[b]	0.0±0.0[b]
2	94.0±12.2[a]	49.1±6.7[a]	0.0±0.0[b]	0.0±0.0[b]	0.0±0.0[b]
3	90.3±16.2[a]	67.3±7.9[a]	0.0±0.0[b]	0.0±0.0[b]	0.0±0.0[b]
4	70.1±3.8[b]	104.3±30.8[a]	0.0±0.0[c]	0.0±0.0[c]	0.0±0.0[c]
5	76.7±11.5[a]	50.3±9.3[a]	0.0±0.0[c]	0.0±0.0[c]	18.8±5.2[b]
6	42.5±7.6[a]	67.1±10.0[a]	0.0±0.0[c]	0.0±0.0[c]	27.2±6.8[b]
7	36.9±4.4[a]	69.8±10.3[a]	8.8±4.1[b]	0.0±0.0[c]	57.7±17.3[a]
8	57.1±10.8[a]	68.4±17.5[a]	14.1±6.2[c]	0.0±0.0[d]	34.1±7.7[b]
9	56.9±7.9[a]	62.9±16.7[a]	24.0±5.1[b]	16.5±4.6[b]	15.4±5.1[b]
10	29.7±5.7[a]	39.8±7.4[a]	15.7±4.2[b]	22.5±5.5[b]	27.0±7.3[ab]
11	35.2±6.3[a]	58.9±14.4[a]	30.9±9.6[ab]	24.5±4.9[bc]	23.3±5.9[bc]
12	42.1±7.6[a]	29.9±5.1[a]	21.5±8.6[b]	23.8±7.6[b]	10.7±3.3[b]

Values followed by identical letters do not differ significantly for comparisons between treatments (columns) within each weekly sample (row), mixed model. Multiple comparisons were subjected to Bonferroni correction ($\alpha = 0.005$). The timepoint zero sample was taken one day before experimental treatments were applied.

Table 2. Mean (±SE) numbers of *Culex* spp. larvae + pupae observed in tires at weekly intervals pre- and post-treatment with insecticides in (A) dry and (B) wet season experiments in southern Mexico.

Sample (weeks)	Treatment				
	Control	VectoBac	1 ppm spinosad	5 ppm spinosad	Temephos
(A) Dry season trial					
-2	133.9±37.3^{a}	72.7±21.9^{ab}	64.3±28.8^{b}	134.2±43.2^{a}	53.3±29.1^{b}
-1	78.7±17.7^{a}	55.1±18.9^{a}	64.1±28.5^{a}	53.3±28.3^{a}	77.3±21.4^{a}
0	92.5±33.1^{a}	51.8±12.3^{a}	112.0±30.4^{a}	28.4±9.7^{b}	31.6±7.2^{ab}
1	65.1±10.2^{a}	22.3±8.6^{b}	0.0±0.0^{c}	0.0±0.0^{c}	0.0±0.0^{c}
2	25.0±9.2^{a}	13.4±4.1^{a}	0.0±0.0^{b}	0.0±0.0^{b}	0.0±0.0^{b}
3	53.3±21.1^{a}	12.9±6.0^{b}	0.0±0.0^{c}	0.0±0.0^{c}	0.0±0.0^{c}
4	74.7±18.0^{a}	27.4±7.4^{b}	0.0±0.0^{c}	0.0±0.0^{c}	31.5±10.9^{b}
5	56.9±17.3^{a}	21.6±10.0^{b}	0.0±0.0^{c}	0.0±0.0^{c}	15.6±6.4^{b}
6	39.9±11.1^{a}	18.6±7.5^{b}	0.0±0.0^{c}	1.0±0.4^{c}	25.3±13.1^{b}
7	66.0±24.6^{a}	17.9±10.2^{b}	1.2±1.2^{c}	0.0±0.0^{c}	6.7±4.4^{bc}
8	88.0±41.8^{a}	13.7±5.1^{a}	4.5±4.2^{b}	0.0±0.0^{b}	38.4±11.6^{a}
9	76.7±13.0^{a}	21.5±5.8^{b}	9.4±4.9^{c}	2.0±1.9^{c}	9.4±3.0^{c}
10	73.1±19.3^{a}	69.0±20.3^{a}	9.2±6.8^{b}	4.1±2.3^{b}	27.1±7.9^{a}
11	110.6±18.3^{a}	12.5±2.0^{b}	30.0±10.6^{b}	1.4±1.4^{c}	115.1±33.4^{a}
12	97.4±18.2^{a}	55.3±19.1^{b}	6.0±4.9^{c}	6.5±5.9^{c}	12.3±2.4^{bc}
(B) Wet season trial					
-1	61.7±18.4^{a}	46.7±8.9^{a}	38.0±9.9^{b}	54.3±11.1^{a}	14.6±3.2^{b}
0	27.3±5.0^{a}	5.3±2.9^{b}	24.2±4.0^{a}	16.4±3.3^{a}	19.6±5.4^{a}
1	15.7±8.0^{b}	24.0±4.8^{a}	0.0±0.0^{c}	0.0±0.0^{c}	0.0±0.0^{c}
2	24.2±5.9^{b}	78.5±26.1^{a}	0.0±0.0^{c}	0.0±0.0^{c}	0.0±0.0^{c}
3	22.5±10.1^{a}	22.4±10.3^{a}	0.0±0.0^{b}	0.0±0.0^{b}	0.0±0.0^{b}
4	70.5±18.4^{a}	14.4±9.2^{b}	0.0±0.0^{c}	0.0±0.0^{c}	12.4±5.5^{b}
5	22.9±7.0^{a}	10.7±2.7^{a}	0.0±0.0^{b}	0.0±0.0^{b}	29.7±20.0^{a}
6	6.2±2.2^{b}	5.1±4.5^{b}	3.5±2.0^{b}	0.0±0.0^{c}	22.9±9.8^{a}
7	10.6±4.8^{a}	18.3±9.9^{a}	0.0±0.0^{b}	0.7±0.7^{b}	15.1±5.7^{a}
8	14.8±4.1^{a}	14.1±4.5^{a}	0.3±0.3^{b}	1.1±1.1^{b}	12.5±4.6^{a}
9	48.5±11.3^{a}	5.1±2.5^{b}	7.4±2.5^{b}	0.4±0.3^{c}	9.5±2.0^{b}
10	4.1±1.7^{c}	13.1±3.0^{ab}	0.0±0.0^{c}	1.9±1.9^{c}	45.3±13.0^{a}
11	5.6±2.0^{a}	9.3±8.3^{a}	2.3±2.3^{b}	0.7±0.7^{b}	0.7±0.7^{b}
12	46.9±11.2^{a}	3.4±1.3^{c}	24.3±8.7^{b}	0.0±0.0^{c}	3.9±2.5^{c}

Values followed by identical letters do not differ significantly for comparisons between treatments (columns) within each weekly sample (row), mixed model. Multiple comparisons were subjected to Bonferroni correction (α = 0.005). The timepoint zero sample was taken one day before experimental treatments were applied.

Table 3. Mean (±SE) numbers of chiromomid larvae + pupae observed in tires at weekly intervals pre- and post-treatment with insecticides in (A) dry and (B) wet season experiments in southern Mexico.

Sample (Weeks)	Treatment				
	Control	VectoBac	1 ppm spinosad	5 ppm spinosad	Temephos
(A) Dry season trial					
-2	0.0±0.0[a]	0.0±0.0[a]	0.0±0.0[a]	0.0±0.0[a]	0.0±0.0[a]
-1	0.0±0.0[a]	0.0±0.0[a]	0.0±0.0[a]	0.0±0.0[a]	0.0±0.0[a]
0	0.0±0.0[a]	0.0±0.0[a]	11.0±8.1[b]	0.0±0.0[a]	0.0±0.0[a]
1	0.0±0.0[a]	0.0±0.0[a]	0.0±0.0[a]	0.0±0.0[a]	0.0±0.0[a]
2	0.0±0.0[a]	0.0±0.0[a]	0.0±0.0[a]	0.0±0.0[a]	0.0±0.0[a]
3	4.3±1.9[b]	15.8±10.8[b]	0.5±0.4[ab]	0.0±0.0[a]	0.0±0.0[a]
4	8.6±4.0[b]	3.1±1.2[ab]	0.0±0.0[a]	0.0±0.0[a]	4.9±1.9[b]
5	22.3±8.1[c]	8.0±3.1[bc]	0.6±0.3[ab]	0.0±0.0[a]	18.7±9.6[c]
6	20.2±9.1[c]	33.7±12.7[c]	11.6±10.8[ab]	1.9±1.0[a]	42.8±16.3[c]
7	33.6±14.7[b]	16.9±5.5[b]	1.3±1.0[a]	0.0±0.0[a]	26.7±5.4[b]
8	14.2±4.6[b]	40.1±30.8[bc]	4.5±2.9[a]	0.3±0.3[a]	48.5±14.1[c]
9	24.7±7.3[c]	28.4±9.7[c]	3.7±1.4[b]	0.3±0.3[a]	21.9±6.8[c]
10	6.3±2.4[a]	50.5±19.9[b]	7.7±2.6[a]	2.5±0.9[a]	70.2±17.3[c]
11	10.2±6.5[b]	20.9±6.9[c]	13.5±3.8[bc]	0.1±0.1[a]	20.5±11.6[c]
12	48.9±22.8[c]	0.9±0.7[a]	19.8±9.6[b]	5.1±5.0[a]	14.0±3.2[b]
(B) Wet season trial					
-1	15.9±8.0[c]	0.0±0.0[a]	11.3±6.8[c]	1.2±1.2[ab]	0.0±0.0[a]
0	24.1±7.1[c]	11.5±5.9[b]	16.2±9.7[b]	0.2±0.2[a]	13.5±2.5[b]
1	21.3±6.2[b]	0.0±0.0[a]	0.0±0.0[a]	0.0±0.0[a]	2.0±0.9[a]
2	6.6±3.3[b]	4.0±2.9[b]	0.1±0.1[a]	0.1±0.1[a]	8.5±8.2[b]
3	42.5±9.9[c]	1.5±1.1[a]	0.0±0.0[a]	0.0±0.0[a]	20.5±6.2[b]
4	52.6±12.4[c]	3.5±1.4[a]	0.2±0.2[a]	0.5±0.4[a]	32.8±11.4[b]
5	40.1±11.9[c]	10.2±2.6[b]	1.2±1.1[a]	0.1±0.1[a]	58.5±18.7[c]
6	6.6±1.5[b]	9.5±3.0[b]	0.5±0.4[a]	0.2±0.1[a]	20.0±11.6[b]
7	2.9±1.3[a]	8.8±6.7[a]	1.7±0.9[a]	2.1±1.0[a]	28.1±9.4[b]
8	22.9±6.5[b]	18.5±8.2[b]	1.2±0.6[a]	2.1±2.1[a]	27.9±6.8[b]
9	7.7±5.6[b]	6.1±2.7[b]	1.1±1.1[a]	5.6±2.7[b]	8.4±1.6[b]
10	20.4±7.0[c]	6.3±1.4[b]	2.9±1.3[a]	5.1±2.5[b]	23.9±7.1[a]
11	5.2±1.1[b]	23.7±11.9[b]	0.0±0.0[a]	2.0±2.0[a]	11.5±3.9[b]
12	4.1±4.1[a]	9.9±2.9[b]	0.0±0.0[a]	1.1±0.8[a]	13.3±3.9[b]

Values followed by identical letters do not differ significantly for comparisons between treatments (columns) within each weekly sample (row), mixed model. Multiple comparisons were subjected to Bonferroni correction (α = 0.005). The timepoint zero sample was taken one day before experimental treatments were applied.
